# Genome-Wide Analysis and Hormone Regulation of Chitin Deacetylases in Silkworm

**DOI:** 10.3390/ijms20071679

**Published:** 2019-04-04

**Authors:** Ziyu Zhang, Jiamin Yan, Qing Liu, Yuhao Zhang, Jing Gong, Yong Hou

**Affiliations:** 1 College of Biotechnology, Southwest University, Chongqing 400715, China; azzy@email.swu.edu.cn (Z.Z.); yanxiaomin@email.swu.edu.cn (J.Y.); lq753159@email.swu.edu.cn (Q.L.); zyh19951014@email.swu.edu.cn (Y.Z.); 03gongjing@163.com (J.G.); 2 Chongqing Engineering and Technology Research Center for Novel Silk Materials, Chongqing 400715, China; 3 Biological Science Research Center, Southwest University, Chongqing 400715, China

**Keywords:** *Bombyx mori*, chitin deacetylases, 20-hydroxyecdysone, juvenile hormone, transcription factors

## Abstract

Chitin deacetylases (CDAs) are a group of enzymes involved in chitin metabolism in insects; they play a critical role in molting, pupation, and the modification of chitin. In this study, we identified several CDAs in the silkworm, *Bombyx mori* (*Bm*CDA), and investigated the effect of various hormones on their expression in *B. mori* larvae and embryo cell lines (*Bm*E). Eight genes encoding *Bm*CDAs were identified in the silkworm genome. They showed different expression patterns in different tissues, and were classified into three types based on where they were expressed: the exoskeleton, digestive organs, and genital organs. Moreover, we found that some *BmCDAs* showed upregulated expression during the molting period, especially during the fourth molting period in larvae. We also verified that the expression of *BmCDA1–6* was upregulated by treatment with 20-hydroxyecdysone not only in larvae, but also in *Bm*E cells. Interestingly, juvenile hormone analog treatment also upregulated the expression of some *BmCDAs*. The overexpression of several transcription factors revealed that the POU transcription factor POUM2 may play a major role in the regulation of *BmCDA* expression. Finally, the silencing of *BmCDA1* and *BmCDA2* did not lead to abnormal phenotypes or death, but may have led to delays in silkworm pupation. These results provide important information about lepidopteran insects in terms of chitin deacetylases and the regulation of their expression.

## 1. Introduction

Chitin is a polysaccharide that is formed by the polymerization of N-acetylglucosamine. It is widely found in the shells of crustaceans, skin of insects, and cytoderm of fungi [1], as well as in many green algae [2]. In animals, chitin mainly functions to support the exoskeleton of the body and protect the organism from the environment outside. Chitosan is obtained by the deacetylation of chitin, and has wide applications in medicine, food, chemicals, cosmetics, biochemistry, and biomedical engineering [3]. In insects, growth and development is majorly associated with the biosynthesis and modification of chitin [4]. The initial substrate in the insect chitin biosynthesis pathway is trehalose—this pathway leads to the synthesis of chitin precursors by chitin synthase [5]. Chitin precursors are partially deacetylated to form chitin, and the deacetylation of chitin is necessary for the structure, permeability, and mechanical properties of the cuticle that makes it more soft and soluble in most insects [6,7,8,9,10].

Chitin deacetylases (CDAs) are a kind of metalloenzyme that belong to the carbohydrate esterase family 4 (CE-4), and chitin deacetylation is catalyzed by CDAs. CDAs were first discovered in the cell walls of fungi [11]. Bacteria and marine bacteria have subsequently been found to contain this enzyme. CDAs have therefore been extensively studied in fungi and bacteria [12]. CDAs have also been detected in many insects, including *Trichoplusia ni* [13], *Tribolium castaneum* [14], *Heliocoverpa armigera* [15], *Mamestra configurata* [16], *Choristoneura fumiferana* [17], *Nilaparvate lugens* [18], *Cnaphalocrocis medinalis* [19], etc. Recent biological and phylogenetic studies of CDA sequences from insects have shown that CDAs can be classified into five groups (I–V) and contain five conserved motifs [6,10,14,18]. The enzyme can modify chitin to different degrees in insects to impart a variety of physical structures or properties to the deacetylated chitin [20,21,22]. *CDAs* are usually expressed in the epidermis, peritrophic membrane (PM), trachea, imaginal wing, and other exoskeleton or internal organs that have strong chitin-binding activity, although some may not contain chitin-binding domains [14,20].

The functional roles of CDAs have been studied in different insects. Group I CDAs in *Drosophila melanogaster*, which include serpentine and vermiform enzymes, limit tube elongation by modulating the physical properties of chitin [21,22]. In *T. castaneum*, group I CDAs affect molting throughout the developmental period, and the silencing of *TcCDA1* and *TcCDA2* can lead to failure in molt or even death [20]. The silencing of *CDAs* in other insects, including *C. fumiferana*, *N. lugens*, and the migratory locust, may lead to abnormal phenotypes and high mortality rates, providing further evidence of the role of CDAs in molting [8,17,18]. The silkworm (*Bombyx mori*) is a model lepidopteran insect with major economic value, and has great potential for development. To date, only *Bm*CDA7 has been studied; it was found to be expressed in the PM of the midgut where it changes the permeability of the PM to prevent microbial infections from mulberries [23].

20-hydroxyecdysone (20E) and juvenile hormone (JH), which are among the most important hormones in metabolous insects, play a crucial role in development, metamorphosis, and reproduction. 20E and JH regulate specific gene expression and initiate cascade reactions by binding to target nuclear receptors including the heterodimer ecdysone receptor and ultraspiracle (EcR/USP) and methoprene-tolerant and taiman (MET/Tai) complexes, respectively [24,25]. In the chitin biosynthesis pathway, 20E and its specific receptors were shown to regulate chitin biosynthesis by inducing the expression of five genes in the lepidopteron insect *Spodotera exigua* [26]. JH was shown to activate the expression of three chitin synthase genes in *Leptinotarsa decemlineata* (*LdChS*) at the early stage of each instar [27]. In the chitin degradation pathway, chitinase activity was regulated as an ecdysone-induced response, and chitinase genes have been shown to be affected by 20E in some lepidopteran insects [27,28]. An additional study reported that the 20E-enhanced expression of *BmCHT5* was closely related to the activity of the transcription factor *Bm*BRCZ4 during metamorphosis in silkworms [29]. So far, there are no studies investigating the hormone-induced expression of chitin deacetylases that modify chitin through deacetylation during insect development.

To better understand the phylogenetic relationships of chitin deacetylases in silkworms compared to those in other species, we identified all the CDAs expressed in silkworms and carried out a comparative and phylogenetic analysis of CDAs originating from fungi, bacteria, marine bacteria, and arthropods. Developmental and tissue-specific expression pattern analyses were performed to improve our understanding of *CDA* genes in *B. mori*. In addition, we extensively studied the regulation of *B. mori CDAs*, and examined the impact of multiple transcription factors on chitin deacetylases in silkworms.

## 2. Results

### 2.1. Identification of Putative CDA Genes in the Silkworm Genome

**Figure 1:**
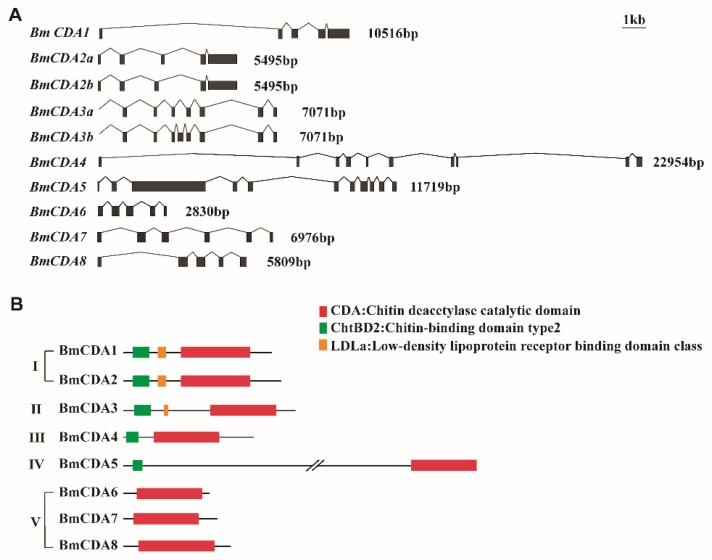
(**A**) Schematic diagram of the exon–intron organizations of eight *Bombyx mori* chitin deacetylase (*BmCDA*) genes. The exons are shown in gray boxes. The lines between the boxes indicate the introns. (**B**) Schematic diagram of the domain architectures of eight *BmCDA* genes. Red boxes indicate chitin deacetylase catalytic domains; green boxes indicate chitin-binding domain type 2; orange boxes indicate low-density lipoprotein receptor binding domain class.

Chitin deacetylases were predicted in the silkworm genome through multiple searches using BLAST. Eight genes encoding CDAs were identified in the *B. mori* genome using the SilkDB and KAIKO databases, and were designated *BmCDA1* to *BmCDA8* (Table 1). Our models also predicted that *BmCDA2* and *BmCDA3* have two alternatively spliced forms: *BmCDA2a* and *BmCDA2b*, and *BmCDA3a* and *BmCDA3b*, respectively (Table 1). The identified genes were different in terms of their sequences, lengths, and number of exons (Figure 1A).

### 2.2. Sequence Analysis of Eight Silkworm CDAs

Based on a previous study of domain architecture, the eight silkworm CDAs were divided into five groups (I–V) [14]. *BmCDA1*, *BmCDA2*, and *BmCDA3*, which were classified as group I and II CDAs, have a CDA catalytic domain, as well as a chitin-binding domain type 2 (ChtBD2) and a low-density lipoprotein receptor (LDLa) binding domain (Figure 1B). *BmCDA4* and *BmCDA5*, which were classified as group III and IV CDAs, respectively, had CDA and ChtBD2 domains, but lacked the LDLa binding domain. Finally, *BmCDA6*, *BmCDA7*, and *BmCDA8*, which were classified as group V CDAs, contained only the CDA domain (Figure 1B).

### 2.3. Phylogenetic Analysis of CDAs

**Figure 2:**
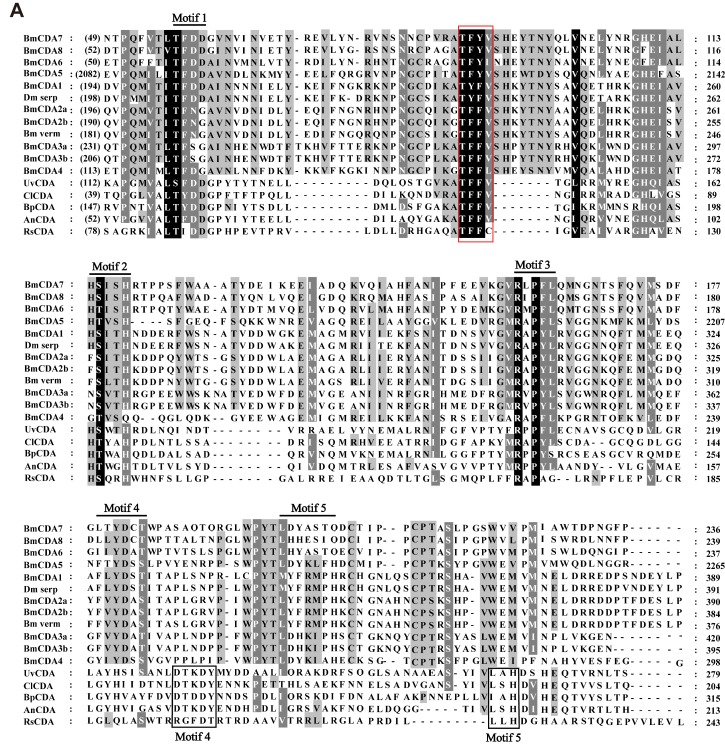

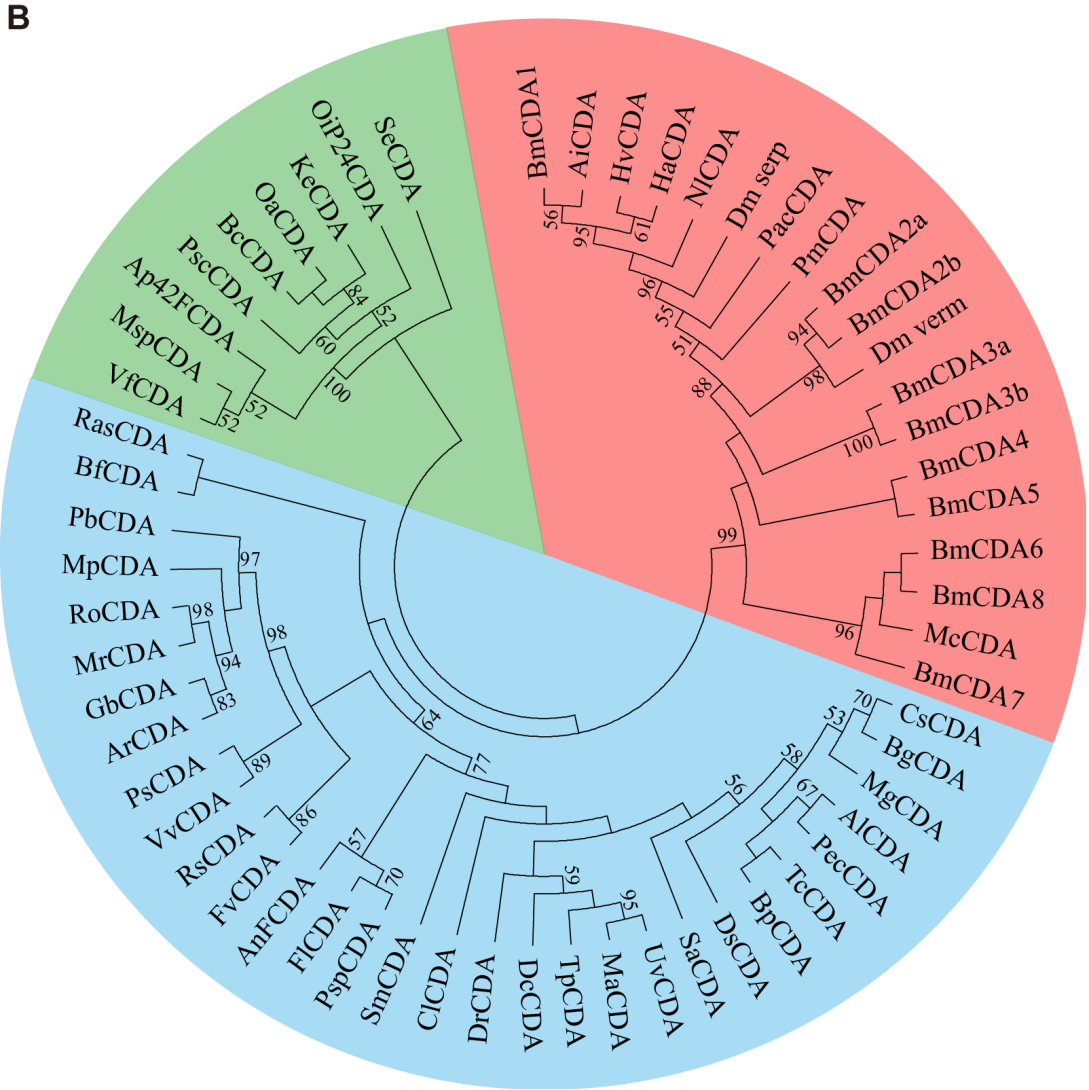
Amino acid sequence alignment and phylogenetic trees of putative chitin deacetylases (CDAs) from fungi, bacteria, and arthropods. (**A**) Amino acid sequence alignment of catalytic domains of carbohydrate esterase family 4 enzymes from fungi, bacteria, and arthropods. The partial amino acid sequences from *Bombyx mori* (Bm), *Ustilaginoidea virens* (Uv), *Colletotrichum lindemuthianum* (Cl), *Blastomyces parvus* (Bp), *Aspergillus nidulans* FGSCA4 (An), and *Ralstonia solanacearum* (Rs) were aligned using CLUSTALX. The conserved motifs (motifs 1–5) are highlighted with black lines and the black boxes represent motif 4–5 for fungi and bacteria. The background of amino acid residues is based on the degree of conservation (black = 100%, dark gray = 80%, light gray = 60%). (**B**) Phylogenetic trees of CDAs from fungi (blue), bacteria (green), and arthropods (red). The partial amino acid sequences from *Bombyx mori* (Bm), *Agrotis ipsilon* (Ai), *Heliothis viriplaca* (Hv), *Helicoverpa armigera* (Ha), *Nilaparvata lugens* (Nl), *Drosophila melanogaster* (Dm), *Panonychus citri* (Pac), *Penaeus monodon* (Pm), *Mamestra configurata* (Mc), *Colletotrichum salicis* (Cs), *Blumeria graminis* (Bg), *Magnaporthe grisea* (Mg), *Aspergillus luchuensis* (Al), *Penicillium chrysogenum* (Pec), *Talaromyces cellulolyticus* (Tc), *Blastomyces parvus* (Bp), *Diplodia seriata* (Ds), *Scedosporium apiospermum* (Sa), *Ustilaginoidea virens* (Uv), *Metarhizium anisopliae* (Ma), *Trichoderma parareesei* (Tp), *Drechmeria coniospora* (Dc), *Diplocarpon rosae* (Dr), *Colletotrichum lindemuthianum* (Cl), *Sphaceloma murrayae* (Sm), *Pestalotiopsis sp.* (Psp), *Fusarium langsethiae* (Fl), *Aspergillus nidulans* FGSCA4 (AnF), *Flammulina velutipes* (Fv), *Rhizoctonia solani* (Rs), *Volvariella volvacea* (Vv), *Puccinia sorghi* (Ps), *Amylomyces rouxii* (Ar), *Gongronella butleri* (Gb), *Mucor racemosus* (Mr), *Rhizopus oryzae* (Ro), *Malassezia pachydermatis* (Mp), *Phycomyces blakesleeanus* (Pb), *Brevibacillus formosus* (Bf), *Ralstonia solanacearum* (Ras), *Vibrio furnissii* (Vf), *Marinomonas sp. MWYL1* (Msp), *Acinetobacter pittii 42F* (Ap42F), *Pseudomonas chlororaphis* (Psc), *Bacillus cereus* (Bc), *Oleispira antarctica RB-8* (Oa), *Komagataeibacter europaeus* (Ke), *Oceanibaculum indicum P24* (OiP24), and *Salmonella enterica* (Se) were aligned using the CLUSTALX. Phylogenetic trees were constructed using MEGA6 software with the maximum likelihood method. A bootstrap analysis of 1000 replications was used and bootstrap values are shown in the cladogram.

The predicted CDA catalytic domain of putative CDA proteins, which belong to the CE-4 family, in insects, fungi, bacteria, and other invertebrates, were analyzed by ClustalX [30]. Multiple sequence alignment analysis showed that all silkworm CDAs contained five conserved motifs (Figure 2A); this was found in most other organisms [31,32]. In addition, three motifs, namely motif 1 (T[F/Y]DD), motif 2 (H[S/T]xxH) and motif 3 (RxPY), were conserved across various species, whereas motif 4 and motif 5 showed major differences depending on the species. Interestingly, we identified a highly conserved area, with a predicted amino acid sequence of threonine-phenylalanine-phenylalanine-valine (TFFV), between motif 1 and motif 2.

To further analyze the homology of the enzymes in these different species, a maximum likelihood (ML) evolutionary tree was constructed (Figure 2B). The ML evolutionary tree was divided into three branches comprising arthropods, fungi, and bacteria (Figure 2B). All eight *B. mori* CDA proteins were within the arthropod clade. *Bm*CDA1 and *Bm*CDA2 were classified into one subclade, and were homologous with the serpentine and vermiform homologues of Drosophila CDAs (*Dmserp* and *Dmverm*), respectively (Figure 2B).*Bm*CDA3, *Bm*CDA4, and *Bm*CDA5 were placed in two separate subclades, distinct from *Bm*CDA1 and *Bm*CDA2 (Figure 2B). *Bm*CDA6, *Bm*CDA7, and *Bm*CDA8 were classified into another subclade that was clearly distinct from the other silkworm CDA proteins (Figure 2B). Furthermore, fungi and bacteria were classified into a separate clade; insects were found to be more relevant to the evolution of fungi than to that of bacteria (Figure 2B).

### 2.4. Tissue and Developmental Expression Patterns of BmCDAs

**Figure 3:**
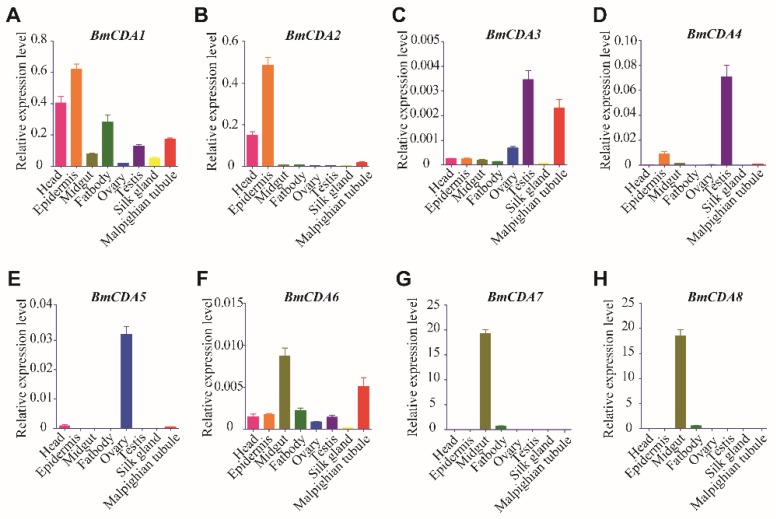
Expression profiles of eight chitin deacetylases (*BmCDAs*) in different tissues of *Bombyx mori*. Total RNA was extracted from different tissues including the head, epidermis, midgut, fat body, ovary, testis, silk gland, and Malpighian tube, which were dissected from larvae at day 3 of the fifth instar.

The tissue expression patterns and developmental expression patterns of eight putative *BmCDAs* were determined by RT-qPCR. The results of tissue expression pattern analysis showed that *BmCDA1* and *BmCDA2*, both of which are group I CDAs, were mainly expressed in the epidermis and head, suggesting that they have functions in the integument (Figure 3A,B). *BmCDA3*, *BmCDA4*, and *BmCDA5*, which are group II, III, and IV CDAs, respectively, were irregularly expressed in the genital organs or Malpighian tube (Figure 3C–E). *BmCDA6*, *BmCDA7*, and *BmCDA8*, which are group V CDAs, were highly expressed in the midgut and seemed to be gut-specific proteins (Figure 3F–H).

**Figure 4:**
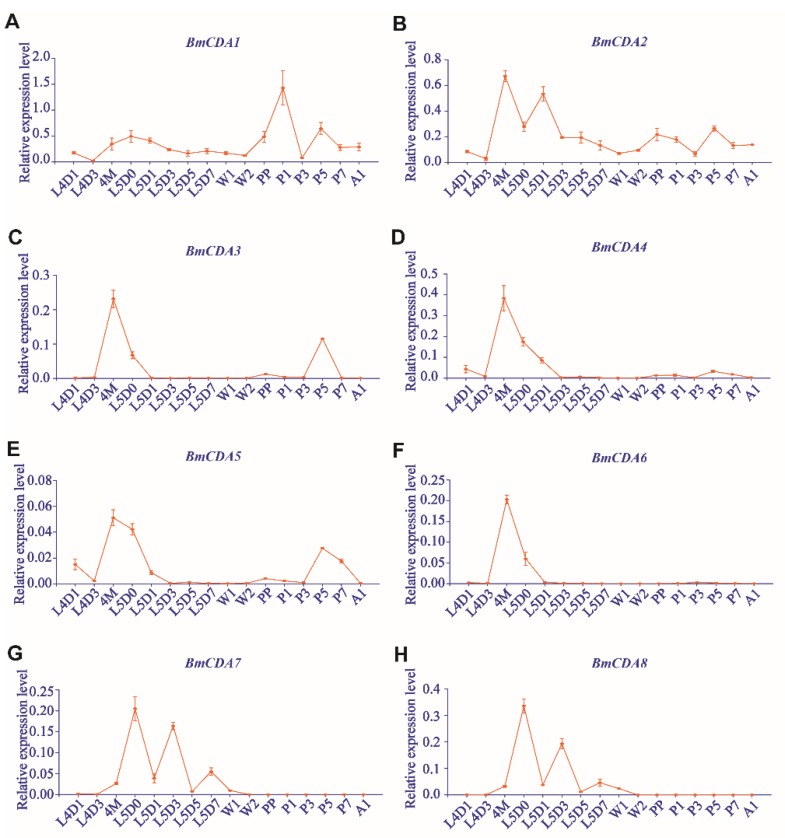
Expression profiles of eight chitin deacetylases (*BmCDAs*) during development in *Bombyx mori*. Total RNA was extracted from whole insects using day 1 of the fourth instar larvae (L4D1), day 3 of the fourth instar larvae (L4D3), molting of fourth instar larvae (4M), newly molted larvae (L5D0), day 1 of fifth instar larvae (L5D1), day 3 of fifth instar larvae (L5D3), day 5 of fifth instar larvae (L5D5), day 7 of fifth instar larvae (L5D7), day 1 of wandering (W1), day 2 of wandering (W2) prepupa (PP), day 1 of pupa (P1), day 3 of pupa (P3), day 5 of pupa (P5), day 7 of pupa (P1), and day 1 of adult (A1).

The analysis of developmental expression patterns showed that the expression levels of *BmCDA1*–*BmCDA6* were significantly increased in the molting stage, and then declined rapidly after molting (Figure 4A–F). Interestingly, the expression of most of them was substantially increased during the fourth larval molting stage, rather than during pupation; this was observed for *BmCDA2*–*BmCDA6* (Figure 4B–F). Only *BmCDA1* was highly expressed during pupation, rather than during larval molting (Figure 4A). *BmCDA7* and *BmCDA8*, the gut-specific group V CDAs, were only expressed during the feeding period (Figure 4G,H).

### 2.5. Influence of 20E on Expression of BmCDAs

**Figure 5:**
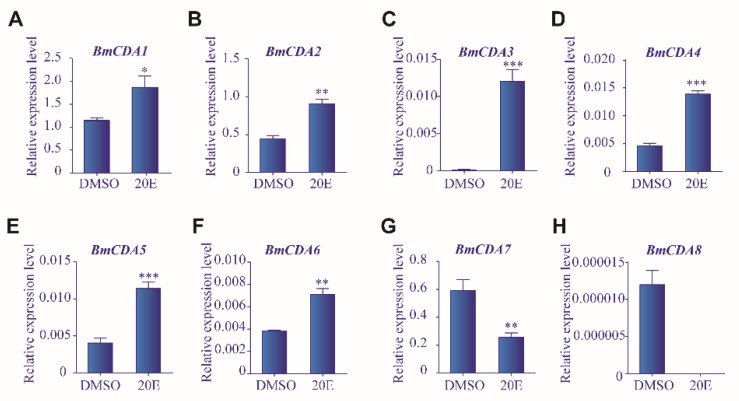
Effect of 20-hydroxyecdysone (20E) on the expression of eight *Bombyx mori* chitin deacetylases (*BmCDAs*). The mRNA expression levels of *BmCDAs* were detected using RT-qPCR after injection of 20E for 24 h. Dimethyl sulfoxide (DMSO) was used in the control group. * *p* < 0.05, ** *p* < 0.01, *** *p* < 0.001 vs. control.

In order to investigate whether the expression of *BmCDAs* was affected by 20E exposure, silkworm larvae treated with 20E were subjected to RT-qPCR. The mRNA expression levels of *BmCDA1*–*BmCDA6* were significantly increased after 20E treatment compared to the control group (Figure 5A–F). However, the expression of *BmCDA7* and *BmCDA8* was decreased after 20E treatment (Figure 5G–H). Furthermore, *BmCDA8* expression was undetectable after treatment with 20E (Figure 5H). Next, *Bm*E cells were treated with 20E. Consistent with our results in silkworm larvae, the mRNA expression levels of *BmCDA1*–*BmCDA6* were increased after 20E treatment compared to the control group (Figure S1). The expression of *BmCDA7* was undetectable, and the expression of *BmCDA8* was significantly decreased after 20E treatment in *Bm*E cells (Figure S1).

### 2.6. Influence of JHA on Expression of BmCDAs

**Figure 6:**
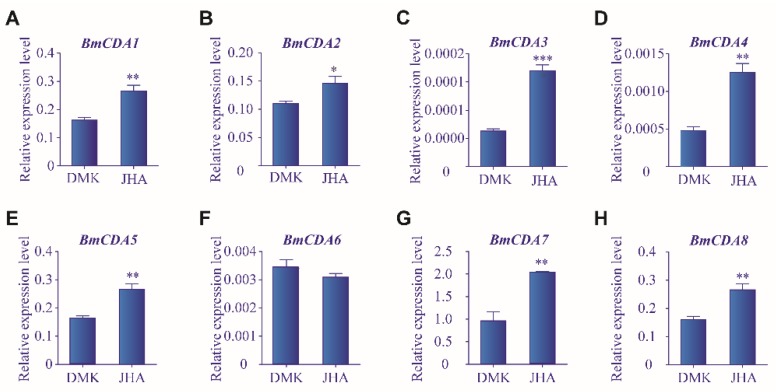
The influence of juvenile hormone analog (JHA) on the expression of eight *Bombyx mori* chitin deacetylases (*BmCDAs*). The mRNA expression levels of *BmCDAs* were detected using RT-qPCR 24 h after injection of JHA. Dimethyl ketone (DMK) was used in the control group. * *p* < 0.05, ** *p* < 0.01, *** *p* < 0.001 vs. control.

In addition to 20E, JH was also used to treat silkworms to analyze its effect on the expression of *BmCDAs*. Most *BmCDAs*, namely *BmCDA1–BmCDA5*, *BmCDA7*, and *BmCDA8* were significantly upregulated after JHA treatment compared to the control group (Figure 6A–E,G,H). *Bm*E cell lines were also treated with JHA. All *BmCDAs* under investigation were upregulated in cells after JHA treatment compared to the control group (Figure S2). Of them, the upregulation of *BmCDA7* and *BmCDA8* was the most significant, with an increase of approximately fourfold. Therefore, it can be concluded that JH plays a crucial role in the regulation of the expression of *Bm*CDAs.

### 2.7. Regulation of Expression of BmCDAs by Overexpression of Transcription Factors

**Figure 7:**
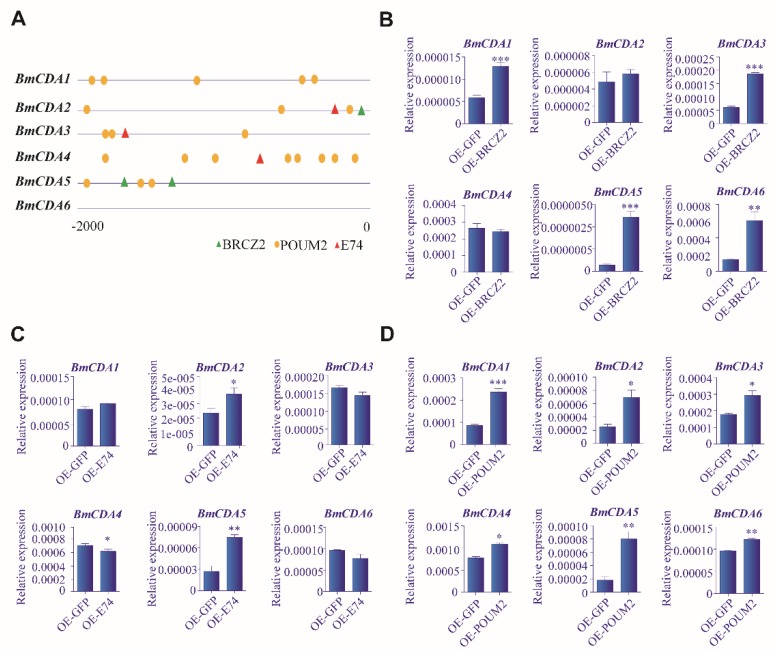
(**A**) Arrangement of the predicted cis-regulatory elements (CREs) in the promoter regions of *BmCDA1–6*. The potential CREs are predicted by MatInspector [33]. (**B**–**D**) Overexpression of BRC-Z2, E74, and POUM2 in *Bombyx mori* embryonic (*Bm*E) cells. Overexpression of (**B**) BRC-Z2, (**C**) E74, and (**D**) POUM2 in *Bm*E cells (OE-BRCZ2, OE-E74, and OE-POUM2) for 72 h, followed by RT-qPCR analysis of *Bombyx mori* chitin deacetylases (*BmCDAs*) gene expression. Overexpression of green fluorescent protein (OE-GFP) was used as the control; error bars represent the standard error of mean (SEM) from three replicates. * *p* < 0.05, ** *p* < 0.01, *** *p* < 0.001 vs. control.

To study the mechanisms of the regulation of *BmCDAs*, six sequences, approximately 2000 bp upstream of each *BmCDA* gene, were analyzed to identify binding sites for several important transcription factors. Many potential transcription factor binding sites were predicted upstream of these genes, including binding sites for broad-complex zinc finger 2 (BRC-Z2), POU transcription factor (POUM2), and Ets transcription factor (E74) (Figure 7A). These three factors were overexpressed by transfecting the relevant vectors into *Bm*E cells. The transcription factors were successfully overexpressed in *Bm*E cells, as shown using RT-qPCR (Figure S3). The expression levels of *BmCDA1*, *BmCDA3*, *BmCDA5*, and *BmCDA6* were found to be upregulated after overexpressing BRC-Z2 in cells (Figure 7B). Furthermore, the expression levels of *BmCDA2* and *BmCDA5* were increased by the overexpressing of E74 in *Bm*E cells (Figure 7C). In addition, the expression levels of *BmCDA1*–*BmCDA6*, which were upregulated by 20E treatment (Figure 5A–F), were also upregulated by the overexpressing of POUM2 in *Bm*E cells (Figure 7D). These results show that the three transcription factors investigated, especially POUM2, potentially played a key role in regulating the expression of *BmCDA*s mediated by 20E.

### 2.8. RNAi of BmCDA1 and BmCDA2

**Figure 8:**
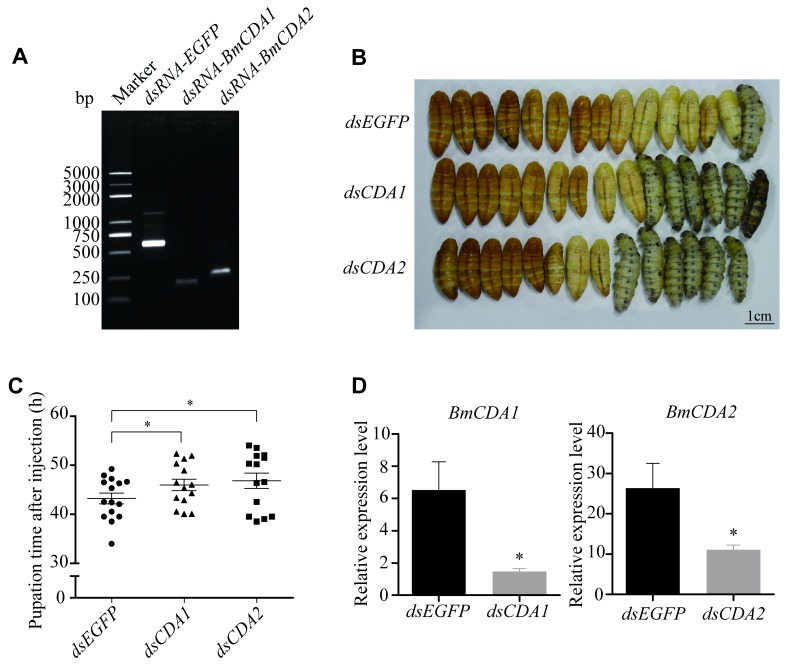
Interference of *BmCDA1* and *BmCDA2* in larvae at the wandering stage. (**A**) Detection of dsRNA from *EGFP*, *BmCDA1*, and *BmCDA2*. (**B**) Phenotypes of insects injected with dsRNA for *BmCDA1* and *BmCDA2*. (**C**) Statistics of pupation time after injection with RNAi. (**D**) Expression levels of *BmCDA1* and *BmCDA2* after RNAi. *dsEGFP* = control.

*BmCDA1* and *BmCDA2* were the most highly expressed *CDAs* in the exoskeletal system. To demonstrate their roles in the molting process of silkworms, dsRNA sequences of *BmCDA1* and *BmCDA2* were injected into larvae. The expression levels of *BmCDA1* and *BmCDA2* were confirmed to be decreased after RNAi (Figure 8D). We then observed the process of metamorphosis from larvae to pupae. The results did not reveal any abnormal phenotypes during pupation, and the structure of the new epidermal tissues was complete and regular. However, the pupation time was delayed after *BmCDA1* and *BmCDA2* RNAi treatment compared to that in the control group (Figure 8B,C).

## 3. Discussion

CDAs have been widely studied in insects by genome-wide analysis, including six *CDAs* from *D. melanogaster*; five each from *Aedes aegypti*, *Anopheles gambiae*, and *Culex quinquefasciatus;* nine from *T. castaneum*; *CDAs* from *Apis mellifera*; and more from a variety of other insects [18]. Alternatively spliced transcripts have also been found for the *CDAs* of insects [8,17,20]. We discovered two alternatively spliced transcripts for both *BmCDA2* and *BmCDA3*, and other alternative splicing forms can potentially be found in other developmental stages or other tissues. In a previous study, different phenotypes were observed after downregulating the two alternatively spliced transcripts of *TcCDA2* [20]. These alternative splice forms in silkworms may also play various roles in different processes and pathways.

In addition to those studied in insects, several microbial CDAs and chitin oligosaccharide deacetylases have been widely studied in fungi and bacteria since 1993 [34,35]. Chitin deacetylation is required during spore wall and cytoderm formation in fungi [36,37]. In the marine bacterium *Vibrio parahaemolyticus*, chitin oligosaccharide deacetylase interacts with chitinase to promote the formation of beta-D-N-acetylglucosaminyl-(1,4)-D-glucosamine (GlcNAc-GlcN) [38]. Fungi, bacteria, and arthropods share a polysaccharide deacetylase catalytic domain, even though they belong to three distinct branches of the evolutionary tree. A previous study has speculated that chitinase was originally obtained from bacteria or baculoviruses [39]. However, we did not find evidence of genetic relatedness or horizontal gene transfer for *CDAs* between bacteria, fungi, and insects.

Based on our sequence analysis, CDAs from fungi, bacteria, and insects share three conserved motifs: motif 1 (TFDD), motif 2 (H[S/T]xxH), and motif 3 (RxPY). In contrast, motif 4 and motif 5 showed major differences depending on the species. In terms of the structure of fungal chitin deacetylase, two aspartic acid residues in motif 1 are considered to interact with zinc, cobalt, and acetate; two histidine residues in motif 2 are also thought to bind to the metal ions. Motif 3 forms one side of the active site groove and has multiple functions; motif 4 forms the other side of the active site groove and tryptophan is thought to be the most critical residue for acetate binding. Motif 5 forms a hydrophobic pocket with a leucine and a histidine residue [31,32]. Interestingly, we found a highly conserved region between motif 1 and motif 2, containing the amino acid sequence TFFV. Based on our observations of the structure of CDA, we found that TFFV was located at the center of the protein. Therefore, we concluded that the conserved TFFV motif may stabilize the protein structure of CDA.

CDA is widely distributed across various insect tissues, including the epidermis, head, trachea, midgut, and Malpighian tube. In silkworms, *BmCDAs* can be divided into three categories according to their tissue expression patterns. The first group, which includes *BmCDA1* and *BmCDA2*, is expressed in the exoskeleton, while the second group is highly expressed in the digestive organ, and includes *BmCDA6*, *BmCDA7*, and *BmCDA8*. The other group is expressed in other internal organs such as the gonads or Malpighian tube. Interestingly, the classification of *BmCDA*s based on their tissue expression patterns was consistent with their distribution on the evolutionary tree. We also found similar expression patterns in other insects such as *T. castaneum* and *N. lugens* [18]. It was suggested that gene differentiation between groups occurs at an earlier stage, while differentiation of genes within a group occurs at a later stage.

Evolutionarily, *BmCDA1* and *BmCDA2*, which belong to group I *CDAs*, were highly expressed in the exoskeletal system. In Coleoptera, Hemiptera, and Lepidoptera, interference of *CDAs* homologous with *BmCDA1* or *BmCDA2* led to shedding failure in old cuticles and eventually to high mortality [17,18,20]. In contrast, in *B. mori*, interference of *BmCDA1* and *BmCDA2* led to a delayed pupation time. We speculated that other *Bm*CDA proteins may have compensated for the reduction in *Bm*CDA1 and *Bm*CDA2 activity.

Silkworms require large quantities of mulberry leaves during the fifth larval stage; the leaves are digested in the midgut. *BmCDA6*, *BmCDA7*, and *BmCDA8* were highly expressed in the midgut, and their expression was only detected during the feeding period. Previous studies have shown that *Bm*CDA7 should protect silkworms from microbial infections from mulberry leaves by modifying and changing the permeability of the PM [23]. We speculated that other *BmCDAs* expressed in the midgut potentially modified and maintained midgut properties to stabilize the metabolism during digestion in the fifth larval stage.

As a holometabolous insect, the silkworm has two different physiological stages: molting and metamorphosis. In this study, most *CDAs* were upregulated during molting and downregulated after ecdysis, particularly *BmCDA1*–*BmCDA6*. Interestingly, most *BmCDAs* showed the highest expression in the fourth molting stage of the larval–pupal metamorphosis period. The distinction between the larval molting stage and pupal metamorphosis was mainly induced by JH. Our analysis of the effects of JHA showed that almost all *BmCDAs* were upregulated in both tissues and cells, indicating that increased *BmCDA* expression was induced by both 20E and JH in the fourth larvae molting period.

It has been reported that *E74* and *BRC* are the primary response genes induced by 20E treatment, due to the EcR/USP complex [40]. BRC-Z4 is an upstream regulatory factor of POUM2, that controls the pupal-specific expression of *BmWCP4* [41]. POUM2 may act together with BRC-Z2 to regulate vitellogenin gene expression in the silkworm [42]. In this study, we found that some *BmCDAs* were upregulated by BRC-Z2 or E74, but all *BmCDAs* that were activated by 20E showed increased expression after POUM2 was overexpressed. Therefore, POUM2 appears to play a crucial role in the regulation of *BmCDAs* induced by 20E. Moreover, we found that the expression of POUM2 was significantly increased after the overexpression of E74 and BRC-Z2 (data not shown). However, the extent to which POUM2 regulated the expression of *BmCDAs* as well as its relationship with other important regulators require future investigation.

In conclusion, we identified eight *CDA* genes in the silkworm in this study. The regulation of these genes by both 20E and JH was studied extensively in the *B. mori CDA* family. Moreover, we examined the impact of multiple transcription factors on silkworm *CDAs*. The results suggested that the transcription factor POUM2 played the most important role in regulating *BmCDA* expression. These results improve our understanding of insect CDAs, and provide a background for further studies on chitosan for industrial, agricultural, and pharmaceutical applications.

## 4. Materials and Methods

### 4.1. Experimental Insects and in Vitro Culture of B. Mori Embryo (BmE) Cells

*B. mori* (Dazao) individuals were provided by the College of Biotechnology at Southwest University (Chongqing, China). Silkworm larvae were reared on fresh mulberry leaves at 25 °C. Larvae at day 3 of the fifth instar (5L3D) were dissected to obtain various tissues for expression analysis, including the head, epidermis, midgut, fat body, ovary, testis, and Malpighian tubule. Larvae between day 1 of the fourth instar (4L1D) and day 1 of adulthood (A1) were ground using a mortar and pestle for developmental expression analysis. Samples were stored at –80 °C for subsequent use. *Bm*E cells were cultured in Grace’s insect cell culture Medium, Gibco (Waltham, MA, USA) supplemented with 10% (*v*/*v*) FBS, Bio Basic Inc. (Toronto, Ontario, Canada).

### 4.2. Identification and Analysis of Silkworm CDA Gene Family

*B. mori* chitin deacetylases (*BmCDAs*) were identified using the NCBI BLAST [43], SilkDB [44], and KAIKO databases [45] based on reported chitin deacetylases in GenBank, including *NlCDA1*, *NlCDA2*, *NlCDA3*, and *NlCDA4* from the brown planthopper [18]. The molecular mass and isoelectric points of the genes were predicted using ExPASy [46]. Conserved domains in the protein sequences were identified using NCBI’s conserved domain database [47]. The chromosome position and exon or intron organization of each *BmCDA* was predicted based on the genome-wide model using a specialized BLAST tool in the KAIKO database.

Protein sequences containing the chitin deacetylase catalytic domain in fungi, bacteria, shellfish, and other insects were obtained from NCBI. Next, multiple sequence alignment analyses for the different homologues mentioned above were constructed using ClustalX and GENEDOC [30,48,49]. MEGA6 software [50] was used for phylogenetic analyses, and a phylogenetic tree was constructed by the maximum likelihood method with 1000 bootstrap tests [51].

### 4.3. Developmental and Tissue Expression Profiles of BmCDA Genes

Total RNA was extracted using TRIzol reagent, Invitrogen (Carlsbad, CA, USA) using tissues from 5L3D larvae, as well as from silkworms between 4L1D and adulthood. cDNA synthesis was carried out using the two-step reaction synthesis system, comprising denatured total RNA and reverse transcription using M-MLV Reverse Transcriptase, Promega (Madison, WI, USA) with 5 μg total RNA for each reaction. Real-time quantitative PCR (RT-qPCR) was performed to analyze the tissue expression and developmental expression patterns of *BmCDAs*. The reaction was performed in a 20-μL reaction volume containing 2 μL 200 ng/μL diluted cDNA, 400 nM each primer, and 10 μL NovoStart SYBR qPCR SuperMix Plus, Novoprotein Scientific Inc. (Shanghai, China) in the real-time PCR system, analytikjena qTOWER 2.2, Analytik Jena (Beijing, China) under the following reaction conditions; 95 °C for 30 s, followed by 40 cycles of 95 °C for 3 s, and 60 °C for 30 s. The gene-specific primers are listed in Table S1 and the expression levels of the target genes were normalized against *sw22934*.

### 4.4. Influence of 20E and Juvenile Hormone Analog (JHA) on the Expression of BmCDA Genes

20E (Sigma Aldrich, St. Louis, MO, USA) was dissolved in dimethyl sulfoxide (DMSO) at a working concentration of 1 μg/μL. Next, 10 μg of 20E solution was injected into the spiracles of 5L3D larvae; pure DMSO was injected into the control group. For JH, juvenile hormone analog (JHA), methoprene, Sigma-Aldrich (Shanghai, China) was used instead of JH in larvae. A total of 10 μg JHA solution, dissolved in dimethyl ketone (DMK), was injected into silkworms, as described for 20E treatment. The same volume of DMK was injected into the control group. After 24 h, the cuticles and midguts of larvae were collected for RNA isolation. Reverse transcription and RT-qPCR analysis of *BmCDA* expression were carried out as described in Section 4.3.

For experiments on *Bm*E cells, a 3-μL solution containing 3 μg 20E or JHA was added to *Bm*E cell lines in a six-well Cell Culture Cluster, Corning Inc. (Corning, NY, USA). After 24 h, cells were collected and total RNA was isolated and reverse transcribed, followed by RT-qPCR to detect the expression of *BmCDAs* at a cellular level as described in Section 4.3.

### 4.5. Overexpression of Transcription Factors Targeting 20E

The promoter sequences (2000 bp) of six *BmCDA* genes were obtained from the SilkDB database [44]. Potential cis-regulatory elements (CREs) were also identified by MatInspector within the six gene promoters [33]. Finally, three elements involved in regulation by ecdysone were selected for the following experiment, namely Broad-complex zinc finger 2 (BRC-Z2), POU transcription factor (POUM2), and Ets transcription factor (E74). The corresponding gene sequences of these transcription factors, *brc-z2*, *e74*, and *poum2*, were inserted to a pSL-1180 plasmid for overexpression. Recombinant plasmids were then transfected into *Bm*E cell lines. The overexpression of green fluorescent protein (GFP) was used as a control. After 72 h, *Bm*E was isolated to detect the expression of *BmCDAs*.

### 4.6. RNAi of BmCDA1 and BmCDA2

Double-stranded RNA (dsRNA) targeting *BmCDA1*, *BmCDA2*, and enhanced green fluorescent protein (EGFP) was synthesized by the T7 RiboMAX Express RNAi System, Promega (Madison, WI, USA) (Table S1). dsRNA sequences (50 µg) were then injected into silkworm larvae at the wandering stage. At 24 h after injection, some larvae were dissected, and RNA was isolated to investigate the expression levels of *BmCDA1* and *BmCDA2*. The rest of the silkworms were observed until pupation, and the time of pupation was recorded and analyzed.

### 4.7. Statistical Analysis

Each experiment was repeated three times independently, and Ct values were recorded for comparative analysis. All values are shown as the mean ± standard deviation from three experiments. Significant differences between treatments were detected using Student’s *t*-test in GraphPad Prism 5 [52], and a value of *p* < 0.05 was considered statistically significant.

## Figures and Tables

**Table 1:** Summary of chitin deacetylases (CDAs) in *Bombyx mori.*

Abbreviation	Gene ID in KAIKObase	Gene ID in SilkDB	Protein ID in NCBI	mRNA Length (nt)	ORF (aa)	Chromosome Position	MW (kDa)	pI
*Bm*CDA1	KWMTBOMO01923	BGIBMGA006213	XP_004929283.1	1620	539	Chr4: 8094528-8105043	61.44	4.87
*Bm*CDA2a	KWMTBOMO01924	BGIBMGA006214	NP_001103795.1	1632	543	Chr4: 8113114-8118608	61.54	5.04
*Bm*CDA2b			NP_001103796.1	1611	537		60.86	5.27
*Bm*CDA3a	KWMTBOMO01369	BGIBMGA008988	XP_021207356.1	1701	567	Chr3: 2479822-2484042	65.74	6.82
*Bm*CDA3b			XP_004931841.2	1626	542		62.67	6.26
*Bm*CDA4	KWMTBOMO07206	BGIBMGA010573	XP_012548585.1	1311	437	Chr12: 6820022-6842885	49.79	4.92
*Bm*CDA5	KWMTBOMO02519	BGIBMGA002696	XP_021207767.1	7245	2415	Chr5: 3321984-3333702	273.6	7.93
*Bm*CDA6	KWMTBOMO16344	BGIBMGA013758	XP_004923454.1	1137	379	Chr28: 789788-792617	43.52	5.06
*Bm*CDA7	KWMTBOMO16345	BGIBMGA013757	XP_004923480.1	1137	379	Chr28: 787560-804535	43	5.19
*Bm*CDA8	KWMTBOMO16346	BGIBMGA013756	XP_004923455.1	1143	381	Chr28: 810851-816660	43.32	6.4

Open reading frame (ORF), isoelectric point (pI) and molecular weight (MW) were predicted using the ExPASy Compute pI/M.W tool.

## Supplementary Materials

Supplementary materials can be found at https://www.mdpi.com/1422-0067/20/7/1679/s1.

## Abbreviations

*Bm*	*Bombyx mori*
CDA	Chitin deacetylase
*Bm*E	Embryo cell lines of *Bombyx mori*
20E	20-hydroxyecdysone
JHA	Juvenile hormone analog
DMSO	Dimethyl sulfoxide
DMK	Dimethyl ketone
OE	Overexpression
GFP	Green fluorescent protein
EGFP	Enhanced green fluorescent protein
