# Supplementary material for: Genome-Wide Analysis and Hormone Regulation of Chitin Deacetylases in Silkworm
Source: Int J Mol Sci. 2019 Apr 4;20(7):1679. doi: 10.3390/ijms20071679 (PMC6480692; doi:10.3390/ijms20071679)
Supplement: Supplementary file 1 [file ijms-20-01679-s001.pdf]

Supplementary Materials: Genome -wide analysis and hormone regulation of chitin deacetylases in silkworm

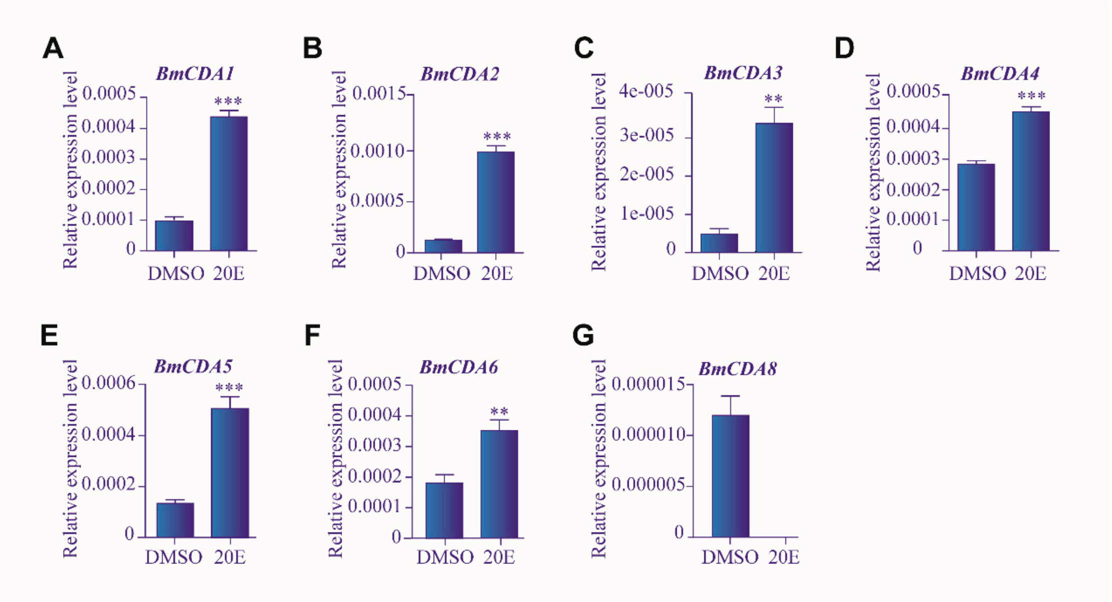

Figure S1. The influence of 20E on the expression of eight *BmCDA*s in *Bm*E cells. The mRNA expression levels of *BmCDAs* were detected using RT-qPCR 24 h after incubation with 20E. DMSO was used for the control group. *$p < 0.05$, **$p < 0.01$, ***$p < 0.001$ vs. control

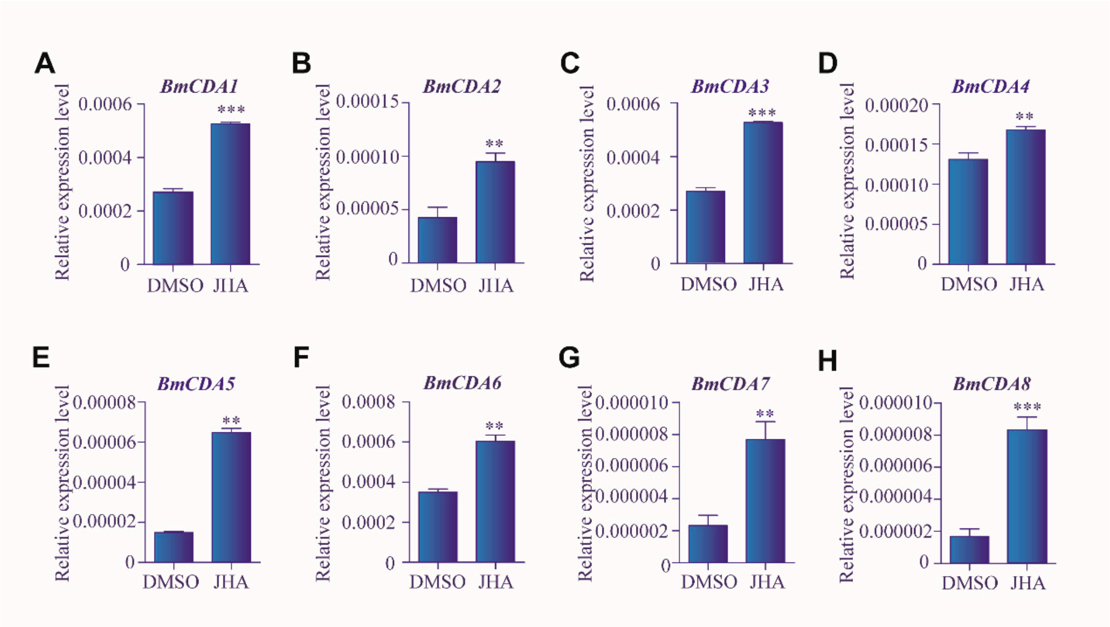

Figure S2. The influence of JHA on the expression of eight *BmCDAs* in *Bm*E cells. The mRNA expression levels of *BmCDAs* were detected using RT-qPCR 24 h after incubation with JHA. DMSO was used for the control group. *$p < 0.05$, **$p < 0.01$, ***$p < 0.001$ vs. control.

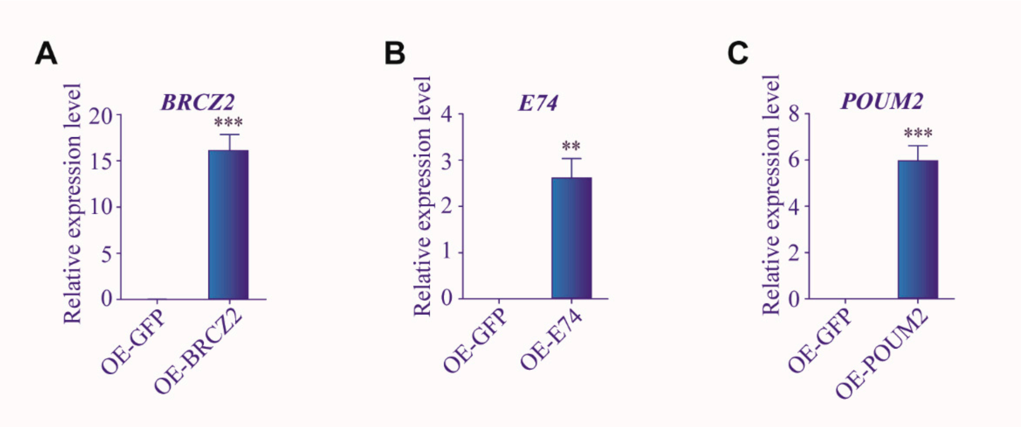

A
BRCZ2
Relative expression level
20
15
10
5
0
***
OE-GFP
OE-BRCZ2
B
E74
Relative expression level
4
3
2
1
0
**
OE-GFP
OE-E74
C
POUM2
Relative expression level
8
6
4
2
0
***
OE-GFP
OE-POUM2

Figure S3. The transcription factors were successfully overexpressed in *Bm*E cells. Overexpression of (B) BRC-Z2, (C) E74, and (D) POUM2 in *Bm*E cells (OE-BRCZ2, OE-E74, OE-POUM2) for 72 h, followed RT-qPCR analysis of *BmCDA* gene expression. Overexpression of green fluorescent protein (OE-GFP) was used as the control; error bars represent the standard error of mean (SEM) from three replicates. *p < 0.05, **p < 0.01, ***p < 0.001 vs. control.

Table S1. Primers for RT-qPCR and dsRNA synthesis.

Table. S1 Primer sequences of different genes

Gene	Primer sequences (5'-3')
BmCDA1-F (RT-qRCR)	TGTGTCCTTCCTGACTGCTTCTG
BmCDA1-R (RT-qRCR)	TGCGATTTCGTGACCCTT
BmCDA2-F (RT-qRCR)	TATTAGTTGGTTCGCTGGTATGC
BmCDA2-R (RT-qRCR)	GCCTGGTGACGCTGTTCTCT
BmCDA3-F (RT-qRCR)	AATGGTCGGCGAAGCAAA
BmCDA3-R (RT-qRCR)	TACGGCCAAAAAGGAGGG
BmCDA4-F (RT-qRCR)	ATAACAGAAGTACCGCTCGATT
BmCDA4-R (RT-qRCR)	AGTTGTTCAAATTTACGGCTCC
BmCDA5-F (RT-qRCR)	AAAACGAATCGGAACACACCCACA
BmCDA5-R (RT-qRCR)	ACAGAGCCAACATTTGCTGGACTT
BmCDA6-F (RT-qRCR)	GCAAATGACAGGAAATTCCAGT
BmCDA6-R (RT-qRCR)	ATAGACCAGGACTCAGAGAAGT
BmCDA7-F (RT-qRCR)	AGGCTGTCGGTTTTGTGCTCTTT
BmCDA7-R (RT-qRCR)	ACCTCACGGTATGTCTCAATGTT
BmCDA8-F (RT-qRCR)	AGGCTGTCGGTTTTGTGCTCTTT
BmCDA8-R (RT-qRCR)	ACCTCACGGTATGTCTCAATGTT
sw22934-F (RT-qRCR)	TTCGTACTGGCTCTTCTCGT
sw22934-R (RT-qRCR)	CAAAGTTGATAGCAATTCCCT
dsRNA-CDA1-F	TAATACGACTCACTATAGGGATGGCGCGCTACGCCCGTGT
dsRNA-CDA1-R	TAATACGACTCACTATAGGGTCGAAGAACAAACCGGCCGG
dsRNA-CDA2-F	TAATACGACTCACTATAGGGTGTTCTGTAATGGTAAACCA
dsRNA-CDA2-R	TAATACGACTCACTATAGGGCAACATTCACAGCACCGTT
dsRNA-EGFP-F	TAATACGACTCACTATAGGGAGACTGAAGTTCATCTGCAC
dsRNA-EGFP-R	TAATACGACTCACTATAGGGAGAGTACAGCTCGTCCATG
